# Baseline immune profiles of local chicken breeds: linking biodiversity, animal health, and vaccination response

**DOI:** 10.1016/j.psj.2025.105565

**Published:** 2025-07-11

**Authors:** Luise Freier, Josefine Stuff, Nina Götzke, Rudolf Preisinger, Christian Grund, Inga Tiemann, Steffen Weigend, Ulrike Blohm

**Affiliations:** a Friedrich-Loeffler-Institut, Institute of Immunology, Suedufer 10, 17493 Greifswald-Insel Riems, Germany; b Precision Livestock Farming, Faculty of Agricultural Sciences and Landscape Architecture, University of Applied Sciences Osnabrueck, Emsweg 3, 49090 Osnabrueck, Germany; c Friedrich-Loeffler-Institut, Institute of Farm Animal Genetics, Hoeltystrasse 10, 31535 Neustadt, Germany; d Lohmann Tierzucht GmbH, Cuxhaven, Am Seedeich 9-11, 27472 Cuxhaven, Germany; e Friedrich-Loeffler-Institut, Institute of Diagnostic Virology, Suedufer 10, 17493 Greifswald-Insel Riems, Germany

**Keywords:** Local chicken breed, Biodiversity, Immune profile, Animal health, Vaccination

## Abstract

Chickens are one of the world’s most important farm animals. With an increasing demand for poultry meat and eggs in recent years, chickens play an essential role in global nutrition and agriculture. However, a severe loss of genetic diversity in livestock has been caused by the focus on high-performance lines, with many traditional and local breeds being threatened with extinction. Although it is assumed that traditional local breeds are more resilient to disease and less susceptible to external influences, little is known about their immunocompetence. This study focuses on the immunological performance of three local chicken breeds (Altsteirer, Bielefelder, and Ramelsloher) in Germany. To evaluate general immunocompetence, blood samples from naïve chicken throughout their lifespan were analyzed by flow cytometry. In adult chickens, minor breed differences were detected regarding the composition of T cell subtypes. However, in day-old chicks the presence of these T cells differs greatly between breeds. To assess the immunological memory after Newcastle Disease Virus vaccination, cellular and humoral immune responses were analyzed. Both, in vivo and in vitro experiments revealed that the duration of immunity depends on the genetic background. Breed-specific proliferation phenotypes were observed up to tolerance induction in one breed after viral re-stimulation. The immune differences among local chicken breeds can explain their differential response to Newcastle Disease vaccination, providing immune markers for breed selection in organic farming. The results of the present study represent the genetic diversity of chicken breeds and show differences in the immunocompetence of local breeds. Thus, this study provides valuable insights into the genetic variation of immunological traits beyond commercial hybrids.

## Introduction

Chickens are vital to global nutrition and agriculture with an increasing demand for poultry meat and eggs (FAO, 2023; Statista, 2025). However, poultry farming faces criticism from consumers and animal welfare advocates, who demand for improvements in husbandry practices, including providing more space and better living conditions, e.g. organic farming (Del Escobedo Bosque et al., 2021). Nevertheless, keeping animals in free-range husbandry systems poses a particular risk of infection through contact with pathogens of wild bird populations (Bonnefous et al., 2022; Crawshaw, 2019; Koch and Elbers, 2006).

Both genetics and husbandry management are fundamental to animal health (Hu et al., 2020; Komlósi, 2022). It is often assumed that traditional local livestock breeds are healthier and better adapted to their environment. However, little is known about the immunocompetence of local breeds. Although commercially bred, high-performance chicken lines dominate global production, traditional and local breeds are becoming increasingly rare. Having been displaced by high-performing specialized hybrids, many of Germany’s native chicken breeds are nowadays endangered and listed on the Red List of Endangered Farm Animal Breeds (German Federal Office for Agriculture and Food, 2025). Of the approximately 8,700 known local livestock breeds globally, only a fraction remained in use today (FAO, 2020). Preserving biodiversity is essential for both human and animal health, as it enhances resilience, reduces susceptibility to external variations, and supports overall well-being (Chen et al., 2024; World Health Organization, 2025). Using local breeds in sustainable agriculture helps maintaining genetic diversity, which is essential for flexible animal breeding in the future.

In general, chicken breeds show considerable variation in genetics and physiology (Mogano et al., 2025; Zhang et al., 2020). These differences also influence immunocompetence, disease resistance and vaccine response. For example, breed-specific susceptibility to influenza viruses has been linked to variation in cytotoxic T cell responses as well as MHC-associated immunity affecting resistance to viral, bacterial and parasitic pathogens (Blohm et al., 2016; Lamont, 1989; Owen et al., 2008). Although immune traits in commercial hybrids are comparatively well studied, the naïve immune status of local chicken breeds remains largely unknown, despite its importance for overall health and effective vaccination.

An important animal disease for which vaccination is mandatory in Germany and other countries is Newcastle Disease Virus (**NDV**). Highly virulent, velogenic NDV strains frequently result in peracute cases characterized by rapid dissemination within the flock, a sudden decline in performance, and a high mortality. The control of Newcastle Disease (**ND**) is governed by the ordinance on prevention of fowl pest and ND. In addition to precautionary measures in the case of an epidemic, this includes mandatory vaccination. In Germany, this obligation is further defined, stating that the owner of a flock of chickens must ensure that ‘sufficient immunity against ND is guaranteed in the entire flock’ (German Federal Ministry of Food and Agriculture, 2005). ND vaccination provides protection against clinical disease and reduces viral shedding. Vaccination with live vaccines induces humoral and cell-mediated immunity, which are both essential for adequate protection (Rauw et al., 2009). The aim of NDV vaccination is to achieve a protective antibody titer in over 85 % of animals in the flock, thereby guaranteeing protection via herd immunity for animals with insufficient titers (van Boven et al., 2008).

There are some data on breed-specific immune responses to ND vaccination with the majority of studies conducted in specific pathogen-free (**SPF**) chickens to facilitate comparing research results (Maas et al., 1999; Mohammad Majid et al., 2023). In both studies, the efficacy of different NDV vaccines was confirmed in SPF chickens. However, Maas et al. (1999) were able to demonstrate the influence of the chicken’s production genetic to ND vaccination, with broilers showing lower antibody titers and less clinical protection than layers. Additionally, a study by Freund et al. (2001) have investigated the duration of humoral immunity after vaccination in specific fancy breeds, finding that maximum antibody titers in fancy chickens are delayed compared to hybrid chicken lines, reaching their peak 7 to 8 weeks after vaccination. They also found differences in the virus neutralization test between breeds, indicating breed-specific susceptibility. Furthermore, recent studies have also shown that genetic factors significantly influence the immune response to ND vaccines in local chicken breeds. The choice of vaccine type and specific genetic variants, such as single nucleotide polymorphisms in the interleukin-4 gene, modulate the response to vaccination. Local genotypes sometimes exhibit different antibody titers and immune profiles after vaccination, suggesting that vaccination strategies should be adapted to local chicken breeds (Edan and Jumaa, 2025; Ushie et al., 2025; Zhou et al., 2024). However, research into the duration of the cellular and, to a certain extent, the humoral immune response in local breeds is still limited.

The aim of this study was to characterize and compare the general naïve immunocompetence at different ages and the immune response to NDV vaccination in three local chicken breeds: Altsteirer, Bielefelder, and Ramelsloher.

## Materials and methods

### Animals and sampling

**Figure 1:**
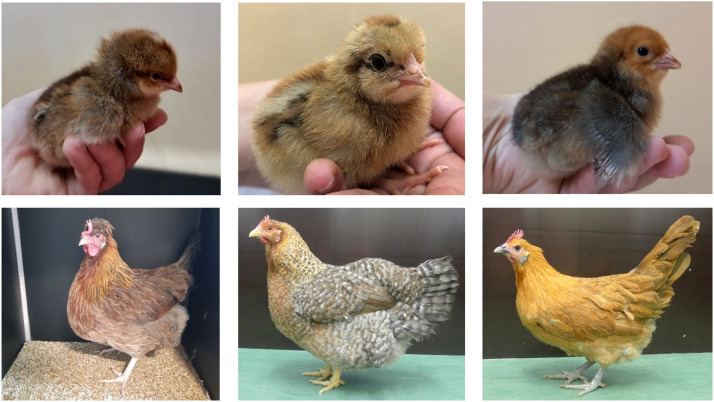
Three local German chicken breeds. Photographs of day-old chicks (upper row) and adult chickens (lower row) show the difference in appearance; from left to right: ALT, BIE, and RAM. ALT = Altsteirer, BIE = Bielefelder, RAM = Ramelsloher.

The three flocks of local chicken breeds Altsteirer (**ALT**), Bielefelder (**BIE**), and Ramelsloher (**RAM**) were established as part of a research project OekoGen, a subproject of the RegioHuhn project funded by the Federal Program for Organic Farming in Germany, which investigates the suitability of these breeds and their crosses with performance genetics of the fattening and laying direction for organic farming. They were selected according to the following criteria: indigenous chicken breeds worth preserving in Germany, they had to be available, reproduce well and already have a certain level of performance. They were considered suitable and - as regionality plays an important role in organic farming - were selected according to their regional distribution, with one breed coming from the north (RAM), one from the center (BIE), and one from the south (ALT) of Germany. As shown in Fig. 1, breeds vary in appearance, both as chicks and as adults. These breeds also differ in performance, as data provided by German Federal Office for Agriculture and Food demonstrate, and are listed on the Red List of native livestock breeds (German Federal Office for Agriculture and Food, 2025).

All three breeds were generated from a parental population at the Campus Frankenforst, the trial farm of the University of Bonn, Germany. The basis of the flocks of the local breeds was a collection of hatching eggs from hobby breeders. Chicks were hatched at the Institute for Animal Welfare and Animal Husbandry of the Friedrich-Loeffler-Institute in Celle, Germany. After hatching, day-old chicks (**DOC**) were transferred to the Friedrich-Loeffler-Institute in Greifswald, Insel Riems, Germany. Table 1 gives an overview on the experimental design of the study.

**Table 1:** Experimental design of the study, Four separate experiments (1, 2, 3a, 3b) were performed to analyze the immunocompetence of the chicken breeds. Given are the age of the tested individuals (DOC = day-old chicks; wk = weeks of age), the category of breed (LB = local breed; CL = commercial line) as well as the number of individuals. NDV = Newcastle Disease.

Experiment(s)	Age	Breeds	Number of individuals per age and breed
1) Development of the juvenile immune system after hatching	DOC, wk1-wk3	LB	10
	wk4	LB	5
2) NDV vaccination	4wk – 12wk	LB	16
3a) Immune system in steady state	6wk	LB	20
	35wk	LB CL	10 10
3b) Immune system in steady state and duration of immunity	wk42, wk50, wk60	LB CL	20 10

To analyze differences in the development of the juvenile immune system after hatching, DOC and juvenile chickens of each breed were housed in their 1st, 2nd, 3rd, 4th, and 6th week of age under conventional conditions at the Friedrich-Loeffler-Institut in Greifswald, Insel Riems, Germany. For each age and breed, 10 animals of both sexes were used. They were fed ad libitum with chick starter feed. To evaluate the immune cell composition in the blood, spleen, and lung, necropsies were performed. For this purpose, the animals were anesthetized with isoflurane inhalation and subsequently euthanized humanly by opening the jugular vein and withdrawing blood. Blood samples were collected in lithium-heparin monovettes (Sarstaedt, Nürnbrecht, Germany). Samples of spleen and lung were taken from freshly culled animals and kept in Phosphate-Buffered-Saline (**PBS**) until further processing in the laboratory.

To investigate the immunocompetence of naïve adult chickens at steady state throughout their lifetime, blood samples from chickens at 35, 42, 50, and 60 weeks of age, provided by project partners at the Institute for Farm Animal Genetics of the Friedrich-Loeffler-Institut, Mariensee, Germany were analyzed. The animal experiment was evaluated following the German law by the responsible ethics committee of the Lower Saxony State Office for Consumer Protection and Food Safety (LAVES) and gained governmental approval under the registration numbers 33.19-42502-05-20A523 and 33.19-42502-04-22-00227. The chickens were kept in aviary keeping. The husbandry conditions were based on Naturland guidelines (4.8 animals per m^2^, 7 hens per nest, 18 cm perch per animal), however daylight and free range could not be provided due to operational reasons. The animals were fed ad libitum with food for laying hens (Legefutter AF ÖTZ 80 TP, Meyerhof zu Bakum GmbH, Melle, Germany). They were vaccinated against NDV with a live attenuated vaccine at 4, 8, and 14 weeks of age and with an inactivated vaccine at 16 weeks of age (Table 2).

**Table 2:** Newcastle Disease Virus vaccination scheme for chickens in animal trial.

Age	Vaccine type
4th week of age	live vaccine
8th week of age	live vaccine
14th week of age	live vaccine
16th week of age	inactivated vaccine

### NDV vaccination

To verify the success of vaccination and to compare the immunological memory after vaccination against NDV under standardized conditions, chicken breeds were vaccinated according to prime and prime/boost vaccination regimens. The procedure was evaluated following the German law by the responsible ethics committee of the State Office of Agriculture, Food safety, and Fishery in Mecklenburg-Western Pomerania (LALLF) and gained governmental approval under the registration number 7221.3-2-003/23. Chickens of the ALT, BIE, and RAM breed were raised under approved biosafety level 2 (BSL2) facility at the Friedrich-Loeffler-Institut in Greifswald, Insel Riems, Germany. After hatching, they were given *ad libitum* access to chick starter feed (PANTO® KAK Kükenalleinkorn, 2 mm pellets, PANTO Ecommerce GmbH, Hamburg, Germany). From the 5th week of age, this starter feed was mixed with a special rearing feed (PANTO® Unikorn, 3 mm pellets, PANTO Ecommerce GmbH, Hamburg, Germany), and from the 7th week of age, the chickens were exclusively fed with this feed.

At 4 weeks of age (d6 after vaccination), 16 chickens of each breed were vaccinated occulonasally (prime vaccination) with Nobilis® ND Clone 30 (Nobilis® ND Clone 30, MSD Tiergesundheit, Intervet Deutschland GmbH, Unterschleißheim, Germany). The live vaccine was administered according to manufacturer's instructions. After a further 4 weeks (d28), half of the prime vaccinated group (*n* = 8) received a second vaccination (boost vaccination) of the vaccine mentioned before. For follow up investigations, blood samples were taken from the wing vein in lithium heparin monovettes (Sarstaedt, Nürnbrecht, Germany). Necropsy for spleen samples was performed 28 days after the boost vaccination (d28 + 28; *n* = 8 per breed per vaccination group). Accordingly, animals were anesthetized by isoflurane inhalation and euthanized by opening the jugular vein and withdrawing blood. Cloacal and oropharyngeal swabs were taken post mortem and stored at - 70°C until further use.

### Peripheral blood mononuclear cell isolation from blood samples and cell staining for flow cytometry analysis

**Figure 2:**
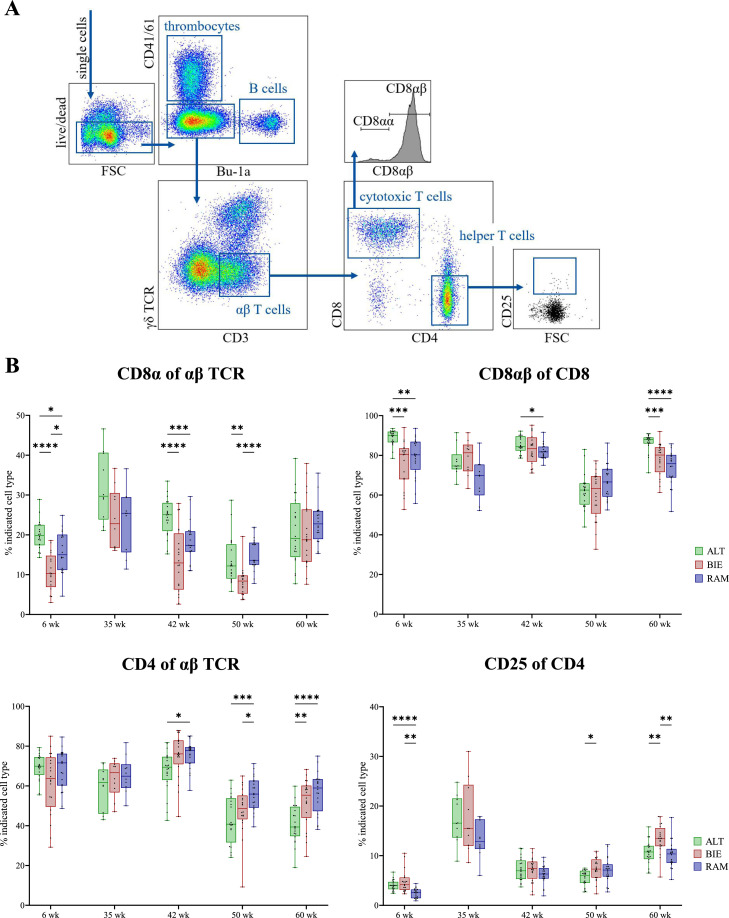
Composition of αβ TCR+ T cell subpopulations in PBMC of naïve chickens. (**A**) Flow cytometry gating scheme for analysis of αβ TCR+ T cells. (**B**) Kinetics of αβ TCR+ T cell subtypes in PBMC of naïve adult chickens from the 6th until the 60th week of age. Upper left: cells expressing CD8α among all αβ TCR expressing cells, upper right: proportion of CD8α+ cells expressing CD8β, lower left: cells expressing CD4 among all αβ TCR expressing cells and lower right: proportion of CD4+ cells expressing CD25. Significance levels were indicated as follows: *P* ≤ 0.05 (*), *P* ≤ 0.01 (**), *P* ≤ 0.001 (***), and *P* ≤ 0.0001 (****). TCR = T cell receptor, PBMC = Peripheral Blood Mononuclear Cells, ALT = Altsteirer, BIE = Bielefelder, RAM = Ramelsloher.

**Figure 3:**
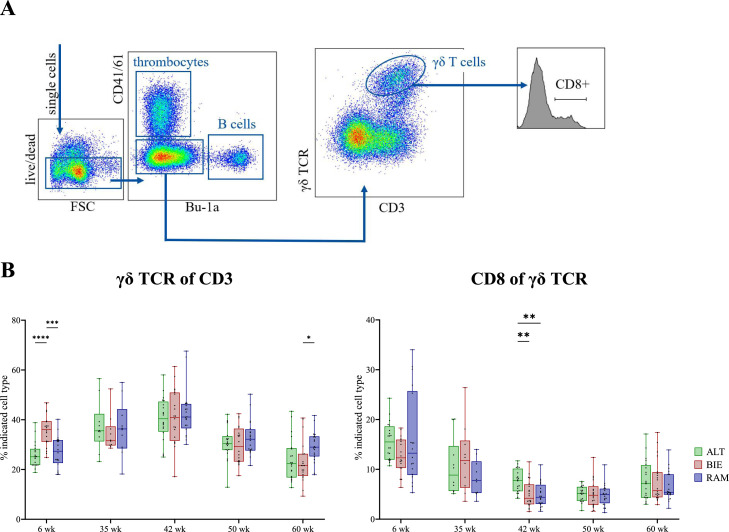
Composition of γδ TCR+ T cell subpopulations. (**A**) Flow cytometry gating scheme for analysis of γδ TCR+ T cells. (**B**) Kinetics of γδ TCR+ T cell subtypes in PBMC of naïve adult chickens from the 6th until the 60th week of age. Left figure: proportion of all γδ TCR expressing T cells among all CD3+ T cells and right figure: proportion of CD8+ γδ T cells. Significance levels were indicated as follows: *P* ≤ 0.05 (*), *P* ≤ 0.01 (**), *P* ≤ 0.001 (***), and *P* ≤ 0.0001 (****). TCR = T cell receptor, PBMC = Peripheral Blood Mononuclear Cells, ALT = Altsteirer, BIE = Bielefelder, RAM = Ramelsloher.

Peripheral Blood Mononuclear Cells (**PBMC**) were isolated by density centrifugation over a Pancoll-gradient (Pancoll human, density: 1.077 g/mL, PAN-Biotech, Aidenbach, Germany). After centrifugation for 30 min at 760 x *g* and room temperature, 1.0 mL of plasma was separated and stored at - 70°C until further use. The interphase containing the PBMC was washed once with PBS. 1 × 10^6^ cells of the cell suspension were used for antibody staining (Table 3) for flow cytometry analysis. The samples were measured by the BD LSRFortessa™ cell analyzer. Compensation and interpretation were done using BD FACSDIVA™ software and FlowJo. The gating schemes are shown in the respective results figures (Figs. 2A, 3A).

**Table 3:** Cell staining for immune cell composition in the steady state.

Primary antibodies				
Antigen	Clone	Conjugate	Manufacturer	Dilution
Chicken CD25	AV142	Pure	Bio-Rad AbD serotec	1:1000
Chicken CD8β	EP42	Pure	Southern Biotech	1:600
Chicken CD4	2-35	Pure	serotec	1:1000
Aqua zombie		V500	Biolegend	1:500
Chicken γδ TCR	TCR-1	FITC	Southern Biotech	1:100
Chicken CD41/CD61	11C3	PE	Bio-Rad AbD serotec	1:20
Chicken Bu-1	AV20	Alexa Fluor 647	Southern Biotech	1:1000
Chicken CD3	CT-3	Pacific blue	Southern Biotech	1:100
Chicken CD8α	CT-8	Biotin	Southern Biotech	1:100

Secondary antibodies				

Antigen	Clone	Conjugate	Manufacturer	Dilution

Mouse IgG1	RMG1-1	BV650	Biolegend	1:500
Mouse IgG2a		PerCP	dianova	1:100
Mouse IgG2b		PE-Cy7	Southern Biotech	1:400
STAV/Streptavidin		BV711	Biolegend	1:200

### Isolation of juvenile leucocytes from blood, spleen and lung samples

To evaluate the kinetics of the immune cell infiltration in immunological compartments, blood, spleen, and lung samples of DOC and juvenile chickens in their 1st, 2nd, 3rd, and 4th week of age were analyzed by flow cytometry for their immune cell composition. PBMC from blood samples were isolated as described before. Spleen and lung samples were collected at necropsy and kept in PBS until they were subsequently processed in the laboratory. Lungs were minced into 2 to 3 mm pieces and digested in 1 mL of digestion mix containing serum-free cell culture medium, Collagenase IV (2 mg/mL, Biochrom, Berlin, Germany) and Type IV DNase I (0.1 mg/mL, Sigma-Aldrich Chemie GmbH, Taufkirchen, Germany) for 1 h at 37°C and 1200 rpm. After incubation, the tissue and debris were removed by filtration through a cell strainer and subsequent washing with PBS for 10 min at 4°C and 350 x *g*. Splenocytes were isolated from spleen samples in a petri dish by carefully pressing them through a fine metal sieve using a stamp together with 10 mL of PBS. The cell suspension was centrifuged for 10 s at 1100 rpm to sediment larger tissue components. Cells in the supernatant were washed by centrifugation for 10 minutes at 350 x *g* and 4°C. After isolation processing, lung and spleen leucocytes as well as PBMC were stained for flow cytometry analysis with chicken-specific commercially validated antibodies (Table 3).

### Re-stimulation and cell staining for flow cytometry analysis

To assess the immunological memory, the proliferation capacity of immune cells after viral in vitro re-stimulation with NDV clone 30 and the velogenic strain NR81/18 was analyzed separately by flow cytometry. For this purpose, blood was centrifuged at 350 x *g* and 4°C for 10 minutes. After centrifugation, 1.0 mL of plasma was separated and stored at - 70°C until further use. Splenocytes from spleen samples were isolated as described before. Together with the cell pellet containing the splenocytes, PBMC from the blood samples were isolated by density centrifugation over a Pancoll-gradient (Pancoll human, density: 1.077 g/mL, PAN-Biotech, Aidenbach, Germany). After centrifugation for 30 min at 760 x *g* and room temperature, the interphase containing the PBMC and the splenocytes, was washed with PBS. 10 to 100 × 10^6^ cells/mL were stained with Tag-it Violet™ (Tag-it Violet™ Proliferation and Cell Tracking Dye, Biolegend, San Diego, CA, United States) according to the manufacturer’s instructions. 4 × 10^6^ cells/mL were cultivated with cell culture medium containing 10 % FCS and 0.05 % β-Mercaptoethanol in a 48 well plate. UV-inactivated NDV strains (clone 30 or NR81/18) were added with a multiplicity of infection of 1 (MOI = 1) and plates were then incubated for 5 days at 38.5°C and 5 % CO_2_. After that incubation period, cells were washed once with PBS and subsequently stained for flow cytometry analysis with chicken-specific commercially available and commonly used antibodies (Table 4). The samples were measured by the BD FACSymphony™ A3 cell analyzer. Compensation and interpretation were done using BD FACSDIVA™ software.

**Table 4:** Cell staining for proliferation after re-stimulation.

Primary antibodies				
Antigen	Clone	Conjugate	Manufacturer	Dilution
Chicken CD4	CT-4	PE	Southern Biotech	1:100
Chicken γδTCR	TCR-1	FITC	Southern Biotech	1:100
Chicken CD8α	CT-8	Biotin	Southern Biotech	1:100
Zombie UV		DAPI	Biolegend	1:500

Secondary antibodies				

Antigen	Clone	Conjugate	Manufacturer	Dilution

Streptavidin		PE-Cy7	Biolegend	1:200

### Evaluation of antibody titer against NDV

Serum samples were analyzed in duplicate by a commercially available enzyme-linked immunosorbent assay (**ELISA**) for the detection of antibodies to NDV according to the manufacturer’s instructions (IDEXX NDV, Newcastle Disease Virus Antibody Test Kit (for use in Chicken), IDEXX Laboratories, Inc., One IDEXX Drive, Westbrook, Maine 04092, USA). Optical density was measured using a Tecan® ELISA reader. As defined by the manufacturer’s guidelines, results with a sample to positive ratio higher than 0.20 were interpreted as positive. Endpoint titers were calculated according to the formula provided by the manufacturer. A titer greater than 396 was considered as positive and indicated vaccination or other exposure to NDV.

### RNA extraction and PCR of swab samples

To evaluate viral shedding after vaccination, viral RNA from swab samples was extracted using the KingFisher® 96 Accessory Kit A and NucleoMag® VET Kit (MACHEREY NAGEL GmbH & Co. KG, Düren, Germany) according to the manufacturer’s instruction. Extraction was performed by KingFisher™ Flex System (Thermo Fisher Scientific, Waltham, MA, USA).

QIAGEN OneStep RT-PCR Kit (QIAGEN GmbH - Germany, Hilden, Germany) was used for ND-M-PCR. The system to detect matrix (M) gene fragments was based on a publication of Wise et al. (2004). Samples were run in a CFX Opus96 Real-Time PCR System (Bio-Rad Laboratories, Hercules, CA, USA). The cycler program started with 30 minutes at 50°C and 15 minutes at 95°C, followed by 40 cycles of 94°C for 30 seconds, 54°C for 30 seconds and 72°C for 30 seconds. Plate read occurred during annealing phase. Samples were run in duplicate with positive control (NDV clone 30, 8.66 × 10^8^ KID_50_) and negative control (RNase-free water). NDV clone 30 (8.66 × 10^8^ KID_50_) was used as standard to calculate virus titers of the samples.

### Statistical analysis

All statistical analyses were performed using GraphPad Prism version 10.4.0 (GraphPad Software, San Diego, CA, USA). Statistical significance was defined at an alpha level of 0.05 (*P* *≤* *0.05*).

Two-way ANOVA was used to analyze the duration of maternal immunity, antibody kinetics after hatching, and NDV vaccination trial data. Tukey’s multiple comparisons test was used for post hoc analysis, except for antibody kinetics after vaccination, where Šídák's multiple comparisons test was applied. Mixed-effects analysis addressed missing values or repeated measures and was used for immune cell kinetics, swab samples, and long-term NDV antibody titers. Group differences were assessed with Tukey’s multiple comparisons test.

Ordinary one-way ANOVA was used for the analysis of T cell re-stimulation and flow cytometry data from DOC and laying hens. Normality and variance homogeneity were assessed using Brown-Forsythe and Bartlett’s tests.

Significance levels were indicated as follows: *P* ≤ 0.05 (*), *P* ≤ 0.01 (**), *P* ≤ 0.001 (***), and *P* ≤ 0.0001 (****).

## Results

### Minor differences in immune cell composition of naïve adult chickens between selected breeds

Leukocytes from blood samples collected from chickens at 6, 35, 42, 50, and 60 weeks of age were analyzed by flow cytometry to evaluate the cell composition in the naïve immune status. Gating scheme focuses on the differentiation of αβ T cells (Fig. 2A) and γδ T cells (Fig. 3A), respectively.

In the first 42 weeks of life, ALT chickens consistently exhibited higher levels of αβ T cell receptor (**TCR**)+ CD8α+ T cells compared to BIE and RAM (Fig. 2B upper left). However, from the 50th week onwards, RAM animals had the highest level of these cells. In the 6th (median = 19.85 %) and 42nd week of age (median = 25.05 %), ALT animals had significantly higher proportions of CD8α+ cells compared to the other two breeds; in the 50th week of age (median = 12.20 %) they had significantly higher proportions than BIE animals. At this age, RAM animals also exhibited significantly higher levels of αβTCR+ CD8α+ T cells (median = 13.60 %) compared to BIE (median = 8.40 %). No significant differences were found at week 35 and 60 in αβTCR CD8α+ T cells. Further analysis of the proportion of conventional cytotoxic CD8αβ+ T cells also revealed breed differences. Except for 35 and 50 weeks of age, ALT had significantly the highest levels of classical CD8αβ+ cytotoxic T cells (Fig. 2B upper right).

From the 42nd week until the 60th week of age, the most prevalent proportion of CD4+ T helper cells were observed in RAM chickens followed by BIE (Fig. 2B lower left). Conversely, ALT exhibited a marked reduction in the proportion of CD4+ T helper cells in comparison to RAM during this period and additionally also to BIE in 50 and 60 weeks of age. At 35 weeks of age, all breeds demonstrated the highest activation status of CD4+ T helper cells measured as CD25+ CD4+ cells (Fig. 2B lower right).

Overall, a notable observation is the decline in TCRαβ+ cell levels irrespective of the breed and in all T cell subpopulations in the 50th week of age. A trend of this decline is already apparent in the 42nd week of age. Thereafter, a fast and complete recovery is seen, with levels increasing again in the 60th week of age. A comparable phenomenon, similar to αβ T cells, can also be observed with γδ T cells (Fig. 3B). In the 60th week of age (concerning γδ T cells; Fig. 3B left) and in the 50th week (concerning CD8+ γδ T cells; Fig. 3B right), all breeds showed a remarkable reduction in the proportion of cells. Nevertheless, in the 6th week of age, BIE animals exhibited a highly significant increase in γδ T cell levels (median = 36.15 %) compared to both ALT (median = 25.10 %) and RAM (median = 27.35 %). However, by the 60th week, RAM animals showed a significantly higher level of γδ T cells (median = 29.00 %) than BIE (median = 21.60 %). At all other time points, only minor, non-significant differences were detected between the breeds. Furthermore, in the 42nd week of age, ALT animals displayed significantly higher proportions of activated, terminal differentiated CD8+ γδ T cells (median = 7.20 %) compared to both BIE (median = 4.20 %) and RAM (median = 4.40 %).

However, when comparing breeds in terms of kinetic over the life span, it should be noted that, due to technical reasons, only 10 animals were available per breed at 35 weeks of age instead of 20 at all other time points.

### At peak laying performance, hens of local breeds had higher levels of CD8+ and CD4+ T cells in blood than high-performance lines

To assess the immunological capacity of laying hens under physiological stress, blood samples were analyzed by flow cytometry at peak laying performance (35 weeks of age). As there were no significant differences in any of the T cell subpopulations detected at 35 weeks of age (Figs. 2B, 3B), the local breeds were combined into one group (distinguishable only by color) and compared with high-performance parental layer (White Rock (**WR**)) and meat-type hens (Ranger (**RG**)), respectively.

**Figure 4:**
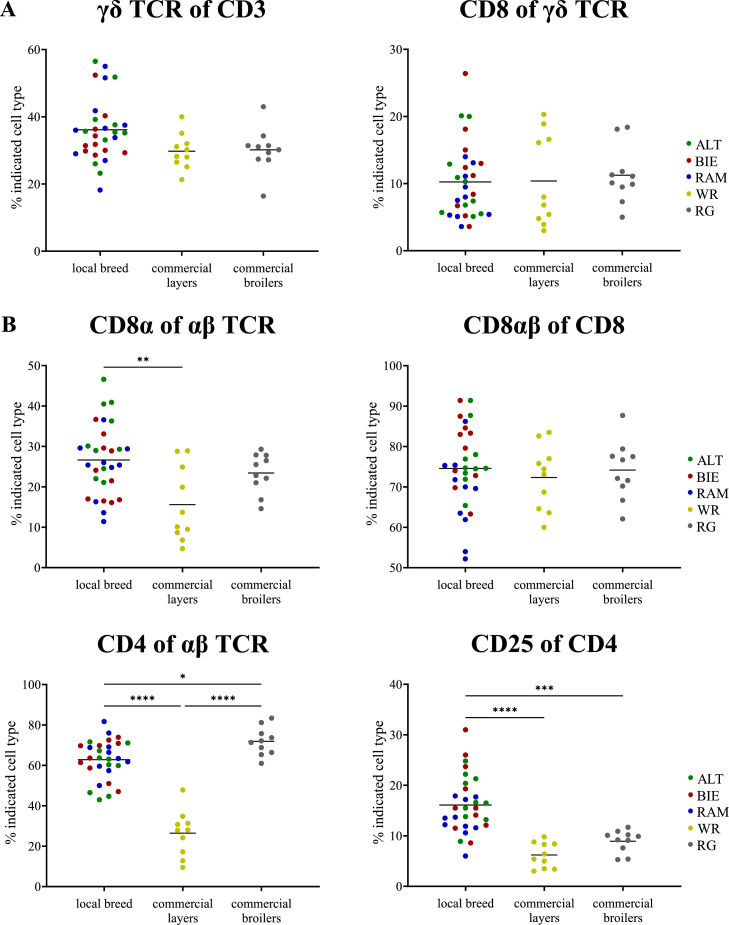
Composition of T cell subpopulations in PBMC of naïve 35-week-old laying hens at peak performance, comparing local breeds and commercial lines. (**A**) γδ TCR+ T cell subtypes. Left figure: proportion of all γδ TCR expressing T cells among all CD3+ T cells and right figure: proportion of CD8+ γδ T cells. (**B**) αβ TCR+ T cell subtypes. Upper left: cells expressing CD8α among all αβ TCR expressing cells, upper right: proportion of CD8α+ cells expressing CD8β, lower left: cells expressing CD4 among all αβ TCR expressing cells and lower right: proportion of CD4+ cells expressing CD25. Significance levels were indicated as follows: *P* ≤ 0.05 (*), *P* ≤ 0.01 (**), *P* ≤ 0.001 (***), and *P* ≤ 0.0001 (****). PBMC = Peripheral Blood Mononuclear Cells, TCR = T cell receptor, ALT = Altsteirer, BIE = Bielefelder, RAM = Ramelsloher, WR = White Rock, RG = Ranger.

Examining CD8+ αβ T cells (Fig. 4B upper left), the animals of all investigated local breeds had, on average, higher levels of CD8α+ cells (mean = 26.64 %). However, comparable levels of conventional cytotoxic CD8αβ+ cells were present in all animals, irrespective of their genetic background (Fig. 4B upper right). Notably, the CD4+ T helper cells from the WR line exhibited a significant reduction in proportion (mean = 26.42 %; Fig. 4B lower left). Nevertheless, these animals displayed an increased proportion of extrathymic CD4+CD8+ double-positive cells within the blood (mean = 35.97 %; Suppl. Fig. S1). In relation to activated CD4+ T helper cells (Fig. 4B lower right), analysis of CD25 expression revealed a higher activation status in the CD4+ cells of animals of the local breeds (mean = 16.09 %). In contrast, regarding γδ T cells, no significant differences were found between local and commercial breeds, neither in their proportions (Fig. 4A left) nor in their activation status as measured by CD8-expressing γδ T cells (Fig. 4A right).

### Significant cellular differences after hatching between selected local breeds

**Figure 5:**
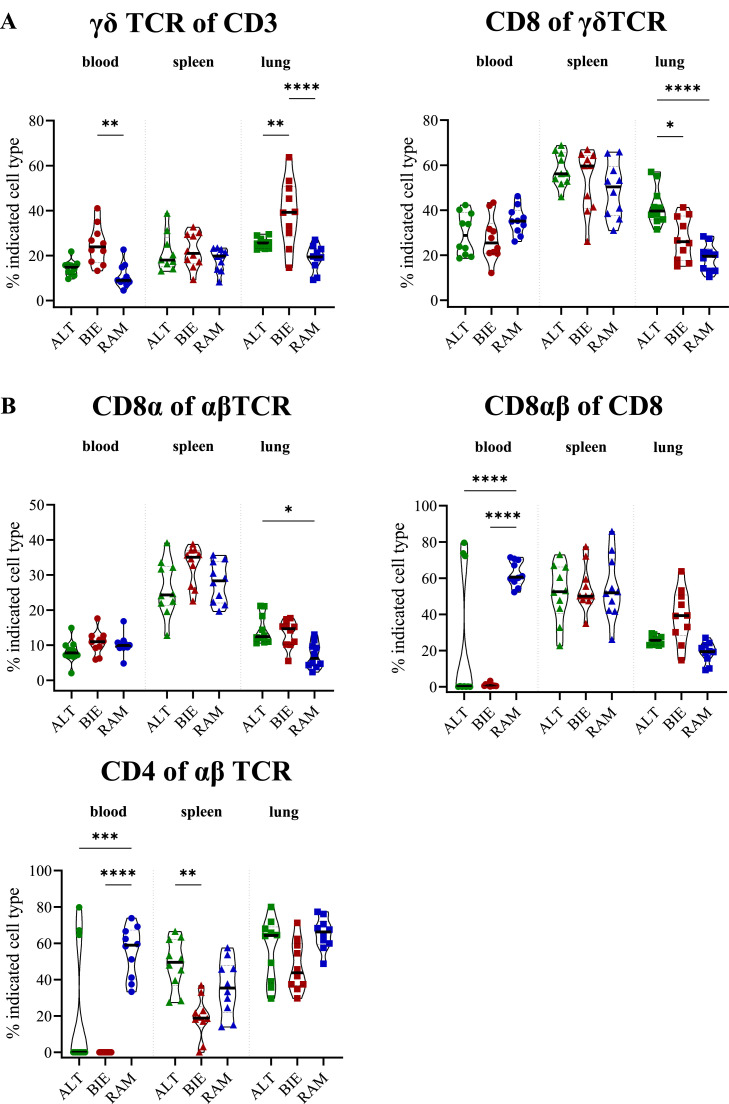
Composition of T cell subpopulations in blood, spleen and lung of day-old chicks (DOC). (**A**) γδ TCR+ T cell subtypes. Left figure: proportion of all γδ TCR expressing T cells among all CD3+ T cells and right figure: proportion of CD8+ γδ T cells. (**B**) αβTCR+ T cell subtypes. Upper left: cells expressing CD8α among all αβ TCR expressing cells, upper right: proportion of CD8α+ cells expressing CD8β and lower left: cells expressing CD4 among all αβ TCR expressing cells. Significance levels were indicated as follows: *P* ≤ 0.05 (*), *P* ≤ 0.01 (**), *P* ≤ 0.001 (***), and *P* ≤ 0.0001 (****). TCR = T cell receptor, ALT = Altsteirer, BIE = Bielefelder, RAM = Ramelsloher.

To investigate whether there are breed specific differences in the kinetics of the development of the juvenile immune system, T cell subpopulations derived from samples of blood, spleen, and lung from DOC and chickens in their 1st, 2nd, and 3rd week of age were analyzed. Interestingly, the greatest differences between the breeds were found in DOC (Fig. 5).

BIE DOC exhibited the highest levels of cells with γδ TCR in both the blood (mean = 23.90 %) and the lung (mean = 39.11 %; Fig. 5A left). Although ALT DOC exhibited significantly lower proportions of γδ T cells in the lung, the highest frequency of activated CD8+ γδ T cells (mean = 41.97 %) was identified in these animals (Fig. 5A right).

The differences in the proportion of cytotoxic CD8αβ+ T cells in the blood were remarkable (Fig. 5B upper right). In RAM DOC, a high proportion of CD8αβ+ T cells (mean = 60.60 %) was observed in all animals. In contrast, in ALT DOC, a comparably high proportion was seen in only some individuals (*n* = 3). However, the majority of ALT DOC exhibited an absence of CD8αβ+ T cells. In BIE DOC, no proportion of CD8αβ+ T cells were detected. A similar result was found for CD4+ T helper cells (Fig. 5B lower left). In this case, all RAM DOC showed high levels (mean = 55.26 %) as well as a few of the ALT DOC (*n* = 3). Conversely, the majority of ALT and all BIE DOC exhibited a complete absence of CD4+ T helper cells. However, ALT DOC showed elevated levels of CD4+ T helper cells in the spleen (mean = 48.35 %). The proportion of CD8α+ αβ T cells was consistent across all breeds, with exception of the lung (Fig. 5B upper left). In this organ, an increased proportion of this T cell subtype was found in ALT DOC (mean = 14.44 %).

From the 1st week of age on, only minor differences between the breeds were detected (data not shown).

### Duration of cellular and humoral immunity against NDV vaccination depends on genetic background

**Figure 6:**
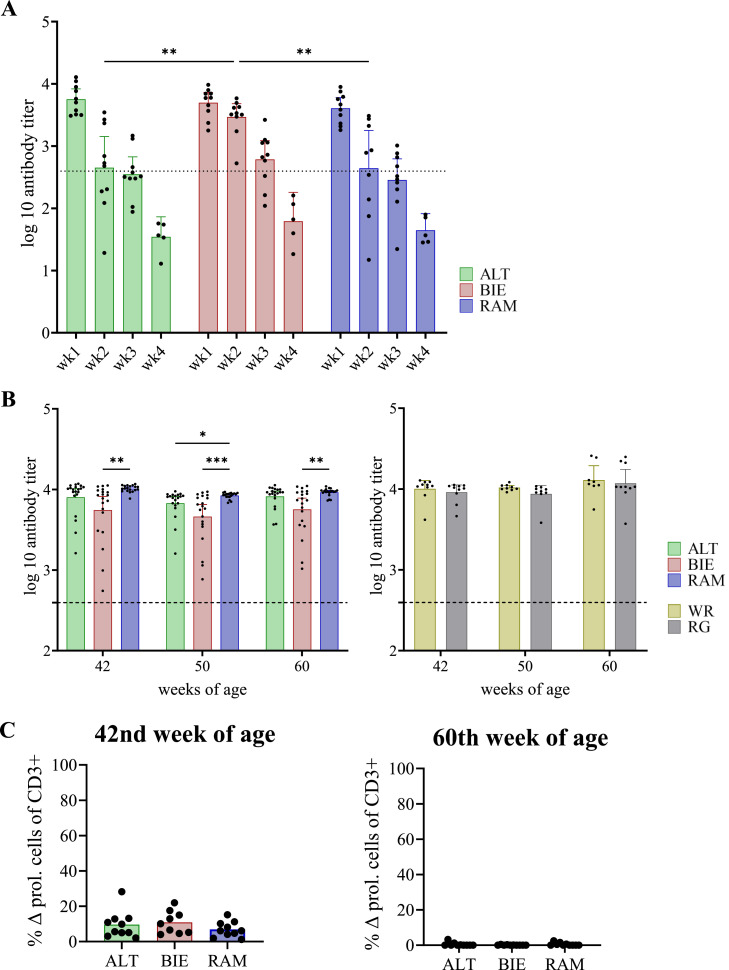
Duration of immunity after Newcastle Disease Virus vaccination. (**A**) Kinetics of maternal antibody titer ex vivo in chicks from the 1st until the 4th week of age. Bars represent the geometric mean; error bars indicate the 95 % confidence interval. (**B**) Duration of humoral immunity: antibody response ex vivo to vaccination at 42, 50, and 60 weeks of age. Left figure: antibody titer in chickens of local breeds (ALT, BIE, RAM) and right figure: antibody titer in chickens of commercial lines (WR, RG). Data are presented as geometric mean with 95 % confidence interval. (**C**) Duration of cellular immunity: T cell response after viral re-stimulation in vitro at 42 (left figure) and 60 (right figure) weeks of age. Significance levels were indicated as follows: *P* ≤ 0.05 (*), *P* ≤ 0.01 (**), and *P* ≤ 0.001 (***). Δ prol. cells = Δ proliferated cells (stimulated – unstimulated control), ALT = Altsteirer, BIE = Bielefelder, RAM = Ramelsloher, WR = White Rock, RG = Ranger.

To evaluate functional differences regarding the immunological memory, the duration of immunity after vaccination against NDV was investigated. Initially, plasma samples of juvenile chickens from the 1st week of age until the 4th week of age were screened for maternal antibodies against NDV by a commercially available ELISA (Fig. 6A). In all breeds, the antibody titers decreased in the period from the 1st week to the 4th week of age. At the latter time point, all animals were negative (antibody titers < 396). In the 2nd week of age, some animals of the ALT (*n* = 3/10) and some of the RAM (*n* = 4/9) were already negative, whereas all BIE animals remained positive for antibodies against NDV. Overall, in this breed, the titer of maternal antibodies decreased slower and less strong compared to ALT and RAM.

Later-on immunological memory was assessed in adult chickens in the 42nd until the 60th week of age. Here, humoral (Fig. 6B) and cellular (Fig. 6C) immune response against NDV after vaccination were investigated.

In adult chickens, BIE animals showed continuously lower antibody titers against NDV and greater individual variation (Fig. 6B left). Strong cluster formation was observed in the RAM, WR, and RG breeds (Fig. 6B left, Fig. 6B right). Except for the WR animals (mean antibody titer WR at 50 weeks of age = 10,449.02), all breeds had the lowest antibody titer at 50 weeks of age (mean antibody titer: ALT = 7,173.93; BIE = 5,597.27; RAM = 8,120.63; RG = 9,026.75). However, throughout the period considered, all animals had antibodies against NDV above the limit. In contrast, the re-stimulability of antigen specific T cells decreased over time. In the 42nd week of age (Fig. 6C left), T cells measured as CD3+ cells, still showed a small ability to proliferate after specific antigen contact (mean Δ proliferated cells of CD3+: ALT = 9.59 %, BIE = 10.89 %, RAM = 6.89 %), but without breed differences. By week 60 (Fig. 6C right), however, proliferation after antigen contact was no longer detectable in any breed.

**Figure 7:**
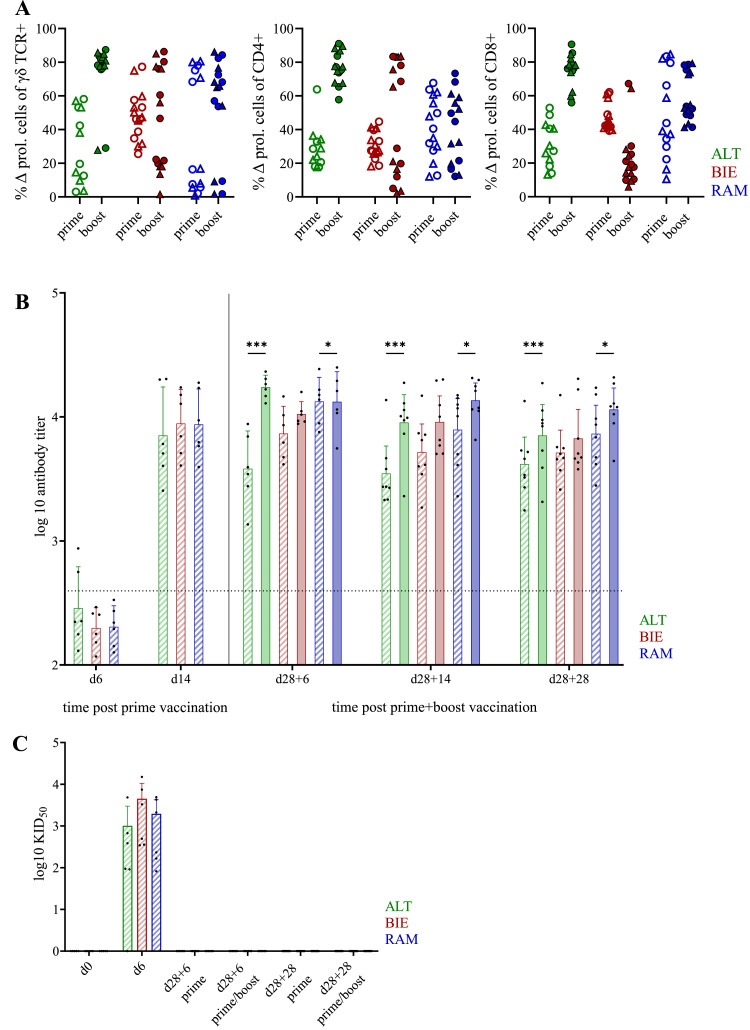
Immune responses to Newcastle Disease Virus vaccination after experimental prime or prime/boost immunization**.** (**A**) Antigen specific cellular response after viral re-stimulation in vitro 28 days after boost vaccination. A 1:1 mixture of PBMC and splenocytes were re-stimulated with vaccine strain NDV clone 30 (circular symbols) or with velogenic strain NDV NR81/18 (triangular symbols). Empty symbols belong to prime group, filled symbols to boost group. Left figure: proportion of proliferated γδ TCR expressing T cells (among CD3+ T cells), middle figure: proportion of proliferated cells expressing CD4 (among αβ TCR expressing cells) and right figure: proportion of proliferated cells expressing CD8α (among αβ TCR expressing cells). (**B**) Antibody response ex vivo in course of the trial. Dashed bars belong to prime group, filled bars to boost group. Data are presented as geometric mean with 95 % confidence interval. (**C**) Oropharyngeal virus shedding. Dashed bars belong to prime group. Significance levels were indicated as follows: *P* ≤ 0.05 (*), *P* ≤ 0.01 (**), and *P* ≤ 0.001 (***). PBMC = Peripheral Blood Mononuclear Cells, Δ prol. cells = Δ proliferated cells (stimulated – unstimulated control), TCR = T cell receptor, ALT = Altsteirer, BIE = Bielefelder, RAM = Ramelsloher.

To facilitate the detection of T cell proliferation after antigen contact, spleen and blood samples were obtained for re-stimulation at earlier points in time after NDV vaccination with different vaccination regimens (prime or prime/boost). Therefore, T cells from spleen and blood samples of vaccinated animals were isolated d28 + 28 and re-stimulated with the vaccine virus NDV clone 30 (Fig. 7A, circular symbols) and as an in vitro challenge with the velogenic NDV strain NR81/18 (Fig. 7A, triangular symbols), respectively. The capability to proliferate was measured by flow cytometry. In ALT animals, higher proportions of proliferated T cells were detected in the boost group, irrespective of the cell type (γδ T cells, Fig. 7A left; CD4+ αβ T cells, Fig. 7A middle; and CD8+ αβ T cells, Fig. 7A right). Regarding BIE animals, in the boost group some animals showed less levels of proliferated cells. The proportion varied between the cell subtypes: concerning γδ T cells and CD4+ αβ T cells, half of the group and concerning CD8+ αβ T cells, nearly all animals showed a decrease of proliferated cells. An increased level of proliferated CD4+ αβ T cells was only observed in some of the BIE animals (*n* = 4). In RAM animals no to minor differences between the two different vaccination groups were detected. Overall, no proliferation differences were found between the vaccine strain NDV clone 30 and the velogenic NDV strain NR81/18.

To compare the specific humoral immune response to NDV between the breeds, the kinetics of antibody titers with the different vaccination regimens were investigated by NDV ELISA (Fig. 7B). After the boost group received the second vaccination, considerable differences between the prime and the boost group were observed in the ALT animals at each time point (d28 + 6, d28 + 14, d28 + 28). In the case of RAM animals, these differences were found to be minor. Conversely, no significant differences were identified in BIE animals. However, the antibody titer against NDV of all animals that received only one or both vaccinations, was above the limit (value > 396) from d14 until the end of the experiment on d28 + 28, across all breeds.

To evaluate the functionality of the anti-NDV antibodies and to assess breed differences in virus shedding, swabs taken post mortem at necropsy were analyzed by quantitative real-time PCR (Fig. 7C). Only 6 days after prime vaccination, the immunized chickens shed the vaccine virus NDV clone 30 oropharyngeally. Swabs from all animals except one ALT chicken were positive for NDV clone 30. Samples from BIE showed the highest viral titer (mean (BIE) = 4,484.83 KID_50_) compared to the other two groups (mean (ALT) = 1,011.33 KID_50_; mean (RAM) = 1,960.17 KID_50_). However, differences between the breeds were not significant. Beyond that, no NDV clone 30 could be detected in oropharyngeal swab samples at later time points (Fig 7A). In contrast, no virus particles were detected in cloacal swabs (data not shown).

## Discussion

The global demand for poultry meat and eggs continues to rise, making it critical to address growing challenges to future livestock production. These include the growing threat of transboundary animal diseases, particularly with regard to Avian Influenza Virus. The virus is spreading worldwide, affecting both wild bird populations and poultry flocks. Additionally, it has been spreading rapidly among dairy cattle in the USA for over a year indicating that transmission to mammals is possible. Due to its zoonotic potential, people who have frequent contact with poultry are at particular risk (Alexakis et al., 2025; Burrough et al., 2024; Halwe et al., 2025). As such, innovative breeding strategies with a focus on immunocompetence and robustness are becoming increasingly important (Neeteson et al., 2023).

Nonetheless, to preserve livestock biodiversity, healthy animals with acceptable productivity need to be developed, for instance by crossing local breeds with high-performance lines (Fiorilla et al., 2023). In the present study, three local breeds were analyzed to assess both their general immune status and suitability as robust crossbreeding partners.

In general, chicks have only a limited level of cellular immunity immediately after hatching and immunocompetence only beginning to develop during the initial weeks of life (Mast and Goddeeris, 1999). Accordingly, in this study, genetically determined differences between chicken breeds in immune cell composition were most pronounced in blood samples taken directly after hatching. Surprisingly, in some blood samples from RAM and ALT DOC, we detected high proportions (i.e. 70 to 80 %) of cytotoxic CD8αβ+ T cells and CD4+ T helper cells. In contrast, a study of SPF White Leghorn DOC by Schmiedeke et al. (2020) demonstrated on average only 40 % of CD8αβ+ T cells in the blood. While the DOC of the RAM breed have an uniformly high proportion of these cells, the ALT chicks clearly form two distinct clusters. Unfortunately, nothing is known about the MHC haplotype of the animals, but it is reasonable to assume that there is MHC diversity in the local breeds, which could explain the differences observed in the ALT. However, although the present study only provides quantitative data on the proportions of immune cells in local breeds, the function of these cells remains unknown. Schmiedeke et al. (2020) transferred naïve lymphocytes derived either from DOC or adult chickens to recipient DOC. Subsequently, infection experiments indicated that only adult not juvenile lymphocytes were able to protect from infection suggesting a deficiency in the immunocompetence of the SPF DOC used in this study. It could be hypothesized that all chicks of the RAM breed and those individuals of the ALT breed, which have high proportions of the discussed T cells, could survive such an infection as chicks. Alternatively, differentiation of the T cells during development could be responsible for the protective effect observed by Schmiedecke et al. (2020). In this case, the mere presence of the cells would not provide protection. As such it will be important in future to expand the findings of the present study to not only provide quantitative data regarding the proportions of immune cells in local breeds, but also analyze the function of these cells.

In analyzing the development of the juvenile immune system, it is also necessary to investigate the transfer and persistence of maternal antibodies. In contrast to mammals, antibody transfer in chickens only occurs via yolk, egg white, and amniotic fluid (Kowalczyk et al., 1985; Kramer and Cho, 1970; Lösch et al., 1986). Maternal antibodies, particularly IgY, are transferred from the hen to the embryo, constituting about 30 % of their immune protection. These antibodies are crucial in early protection against pathogens in immunologically immature newly hatched chicks (Haems et al., 2024; Hamal et al., 2006; Liu et al., 2023). In our study, the mean antibody titer was above the indicated limit value until the 3rd week of age in all three breeds. This is in contrast with most of the results in literature. For instance, Gharaibeh and Mahmoud (2013) demonstrated maternal antibodies to NDV were undetectable in commercial broiler lines by 10 days of age, whereas other studies have reported a loss at 2nd to 4th weeks of age (Ahmed and Akhter, 2003; Hamal et al., 2006; Sahin et al., 2001).

In addition, we detected breed-specific differences in the kinetics of maternal antibody levels against NDV during the first four weeks of age with antibody titers decreasing faster in ALT and RAM chicks compared to BIE. These findings provide important insights into the development of the chick’s own immune system, including the breed-specificity found. The chicks exhibit a sequential synthesis of immunoglobulins, as well as the catabolism of maternal antibodies, leading to the gradual replacement of passive maternal immunity by the chick’s endogenous immune response (Hamal et al., 2006; Niewiesk, 2014). Consequently, it can be deduced that the longer maternal antibodies are detectable, the less the chick’s own, endogenous immunity has developed. In concrete terms, our observations are indicative of a slower development of the endogenous immune system in BIE chicks compared to the other two breeds.

Later in life, only minor differences in immune cell composition between the breeds were detected. It is not clear, if these findings are genetically determined or due to other influences encountered throughout the life span, i.e. nutrition, housing, environmental stress, and social stress, all of which can have an influence on the avian immune system (Dalgaard et al., 2022; Dietert et al., 1994; Hofmann et al., 2020; Morgan and Tromborg, 2007). With increasing age, the expansion of antigen-specific αβ T cells leads to a shift in the γδ to αβ T cells ratio in favor of αβ T cells. On average, we found that ALT chickens had more CD8+ αβTCR+ cells, including CD8αβ+ cytotoxic T cells. The protective function of CD8+ T cells has been shown to contribute significantly to the elimination of several infectious viruses of chickens, including Infectious Bronchitis Virus, Infectious Bursal Disease Virus, Marek’s Disease Virus, and Avian Influenza Virus (Kapczynski et al., 2011; Rauf et al., 2011, 2012; Sarson et al., 2008; Wang et al., 2006). Higher levels of CD8αβ T cells might result in stronger and faster cytotoxic T cell responses, and thus more efficient elimination of virus infected cells. In contrast, RAM chickens exhibited on average more CD4+ αβTCR+ T helper cells. This T cell subpopulation is mainly responsible for assisting both CD8+ T cells and B cells in their specific immune functions (Arstila et al., 1994). For RAM chickens, this could facilitate a stronger and faster adaptive immune response, better control of chronic infections, improved immune regulation, and less susceptibility to diseases. However, it is important to note that a definitive statement about the function and efficacy of the detected cell populations cannot be made at this time.

Only little is known about the naïve immune cell composition of chickens over their life span. Schmucker et al. (2021) analyzed the numbers of immune cell subtypes of the two genetically distinct high-performance layer strains Lohmann Brown-Classic and Lohmann LSL-Classic from the 9th week of age until the 59th week of age. They also detected small differences between the strains; however, patterns of age-related immunological changes were similar. For instance, they reported increased numbers of γδ T cells until the 15th week of age which then constantly decreased during the laying period. In the present study, we also observed an increase in γδ T cells followed by a decrease; however, this was observed at later ages (i.e. 42nd week of age). Furthermore, in contrast to the present study, the authors found low numbers of cytotoxic T cells and CD4+ T helper cells in the blood during the laying period (Schmucker et al., 2021).

In general, the lowest level in all T cell subtypes were observed at week of age 50 regardless of breed. This phenomenon might be probably explained by the fact that chickens molt at this age. Indeed, there is some evidence suggesting that molting contributes to systemic diseases and impairs the cellular immune system in chickens (Alodan and Mashaly, 1999; Holt and Porter, 1992). Specifically, the stress caused by fasting during molting increases circulating adrenal corticosteroid levels (Akram et al., 2002; Latshaw, 1991). This may contribute to an impaired immune response, resulting in a reduction in the number of circulating leukocytes and a reduced splenic leukocyte population. However, it is important to note that chickens generally exhibit a period of recovery following the molt, during which the immune system undergoes a process of strengthening and resilience (Holt, 1992; Holt and Porter, 1992). This is consistent with our results demonstrating that the proportion of all immune cell subtypes increases again in the 60th week of age. Consistent with these results, a study by Kowalczyk et al. (2020) showed that immune cell numbers in the blood of turkeys decrease at the end of the laying period, which they concluded might be responsible for a higher susceptibility to disease.

We initially hypothesized that different breeds would exhibit greater differences at their peak of laying performance because performance is associated with stress, and stress resilience is partly genetically determined (Cahill et al., 2022). Further, immune function and laying performance are both energy-demanding processes that must be maintained in physiological balance with each other, further suggesting that immune function might be compromised by increased reproductive activity (Demas and Nelson, 2011; Knowles et al., 2009). However, the analysis of blood samples from hens at peak laying performance revealed no significant differences among local breeds with the exception of lower CD8+ cytotoxic T cell levels and significantly decreased CD4+ T helper cell levels in the laying hybrids. This is consistent with what was observed by Schmucker et al. (2021) in their two high-performance laying strains. Nevertheless, the laying hybrids using in the present study showed more CD4+CD8+ double positive T cells. A study by Luhtala et al. (1997) has also identified a high proportion of double positive cells in some chicken lines. This cell population has been suggested to play an immunoregulatory role (Dai et al., 2021). Research conducted on different species, particularly humans, have shown that peripheral CD4+CD8+ double positive T cells are functionally mature effector cells with memory functions and may also be actively involved in antiviral immune responses (Nascimbeni et al., 2004). Although direct studies in chickens are limited, these findings suggest that CD4+CD8+ double positive T cells may also play a significant role in the avian immune response; however, further research is needed to understand their exact function and importance in chickens.

Humoral and cellular factors were analyzed in relation to the duration of immunity, generated in response to NDV vaccination. We were able to show that all vaccinated animals had detectable antibody titers to NDV after vaccination at 42, 50, and 60 weeks of age, although with small differences. Neutralizing antibodies play a pivotal role in protection against NDV (Kapczynski et al., 2013; Reynolds and Maraqa, 2000a, 2000b). Whereas antibody titers were found to be elevated in all animals within the commercial breeds and the RAM group, substantial individual variation exists in the BIE group suggesting a potential risk at the individual animal level. A genetically dependent immune response to vaccination was also found by Maas et al. (1999) who showed that broilers had lower antibody titers and were less protected compared to laying hens.

In the present study, antigen-specific T cell responses following viral re-stimulation at 42 weeks of age were minimal and likely biologically insignificant, with no significant differences between the breeds. This response further diminished to below the detection limit by 60 weeks of age. The chickens of the present study were last vaccinated with a live attenuated vaccine at 14 weeks of age, indicating that the duration of immunity based on T cell response must be shorter than 20 weeks. This supports a decisive role of neutralizing antibodies in immunity against NDV with the cellular response being of secondary importance (Kapczynski et al., 2013; Reynolds and Maraqa, 2000a, 2000b).

Our standardized vaccination protocols have revealed breed-specific variations in humoral immune responsiveness, which were influenced by the immunization strategy (prime or prime/boost), indicative of genetically determined differences in adaptive immunocompetence among the examined chicken breeds. Consistent with the findings from the long-term trial, antibodies exceeding the limit value were measured in all investigated animals from 14 days after the initial vaccination. A study by Allan et al. (1978) has demonstrated that hybrid chicken lines reach maximum antibody titers 2 to 4 weeks after vaccination. According to Freund et al. (2001), the antibody peak in fancy breeds was generally delayed to 7 to 8 weeks after vaccination. In the prime vaccinated group of our study, the highest antibody titers in ALT and BIE chickens were measured 2 weeks after vaccination (d14) which is consistent with the results from Allan et al. (1978). In the study by Freund et al. (2001), the BIE chickens were the only fancy breed that showed peak antibody titers 4 weeks after vaccination. However, this is delayed compared to our results. In contrast, similar to what was generally reported for fancy breeds by Freund et al. (2001), RAM chickens had the highest titers only 5 weeks after vaccination (d28 + 6). From a functional standpoint, the absence of viral RNA in swabs taken 4 weeks post-prime and 6 days post-boost supports the presence of protective antibody levels. With respect to the cellular immune response, only chickens in the ALT group benefited from a second vaccination dose. The ALT chickens also showed the strongest increase in antibody levels after boosting, suggesting they benefited the most from receiving a booster vaccination. This is consistent with the results from Dalgaard et al. (2010) using two inbred chicken lines where they showed antigen-specific proliferation of peripheral CD4+ and CD8+ T cells after vaccination with an NDV live vaccine, as well as higher frequencies of circulating γδ T cells after vaccination. In addition, one of their two inbred lines demonstrated a higher proliferation capacity, suggesting genetic differences in these responses. In our study, animals in the RAM group demonstrated only a CD8+ cytotoxic T cell response, whereas γδ T cells and CD4+ T helper cells in the prime and the boost group exhibited comparable reactions. Furthermore, within this group, there was a clear distinction between responders and non-responders among the γδ T cells. Chickens in the BIE group did not demonstrate a beneficial response to the second immunization, suggesting the unfavorable induction of immunological tolerance in CD4+ and CD8+ T cells by the booster vaccination. Immunological tolerance may develop due to breed-specific suboptimal vaccination practices and should be considered in the development of vaccine programs to ensure the effectiveness of vaccination. For instance, Dortmans et al. (2014) detected insufficient immunity to NDV in field-vaccinated broilers which they attributed to inappropriate vaccination practices.

Overall, minor differences in immune cell composition were observed between local breeds throughout their lifespan. However, the greatest differences were seen after hatching, with RAM DOC already having T cell levels comparable to those of adult chickens. The titer of maternal NDV antibodies decreased most slowly in BIE chicks. At peak laying performance, hens of local breeds had higher levels of CD8+ and CD4+ T cells in blood compared to commercial lines. ALT chickens benefited the most from booster immunization, whereas induction of tolerance was observed in BIE.

Our findings clearly demonstrate considerable immunological variation among chicken breeds, emphasizing that a one-size-fits-all approach to vaccination is no longer sufficient. Consequently, immunocompetence needs to be more carefully considered as a breeding criterion in future. The workflow developed in this study provides a basis for standardized breed comparisons and also enables the implementation of breed-specific vaccination protocols and well-founded breeding strategies. Ultimately, this approach supports sustainable poultry farming by combining genetic diversity and animal health.

## Funding

The project is part of the project OekoGen and supported by funds of the Federal Ministry of Agriculture, Food and Regional Identity (BMLEH) based on a decision of the Parliament of the Federal Republic of Germany via the Federal Office for Agriculture and Food (BLE) under the research and innovation program ‘Climate Protection in Agriculture’ (FKZ: 2819OE164).

## CRediT authorship contribution statement

**Luise Freier:** Data curation, Formal analysis, Investigation, Methodology, Visualization, Writing – original draft, Writing – review & editing. **Josefine Stuff:** Data curation, Writing – review & editing. **Nina Götzke:** Data curation, Writing – review & editing. **Rudolf Preisinger:** Data curation, Resources. **Christian Grund:** Data curation, Methodology, Resources. **Inga Tiemann:** Data curation, Funding acquisition, Resources, Writing – review & editing. **Steffen Weigend:** Conceptualization, Data curation, Funding acquisition, Investigation, Resources, Supervision, Writing – review & editing. **Ulrike Blohm:** Conceptualization, Data curation, Formal analysis, Funding acquisition, Methodology, Resources, Supervision, Visualization, Writing – original draft, Writing – review & editing.

## Disclosures

The authors declare that they have no known competing financial interests or personal relationships that could have appeared to influence the work reported in this paper.

## Appendix Supplementary materials

Supplementary Fig. S1 Levels of CD4+CD8+ double positive αβ TCR+ T cells in 35-week-old laying hens at peak performance, comparing local breeds and commercial lines. Significance levels were indicated as follows: P ≤ 0.0001 (****). TCR = T cell receptor, ALT = Altsteirer, BIE = Bielefelder, RAM = Ramelsloher, WR = White Rock, RG = Ranger. Image, application 1
