# Supplementary figures and images for: Baseline immune profiles of local chicken breeds: linking biodiversity, animal health, and vaccination response
Source: Poult Sci. 2025 Jul 11;104(10):105565. doi: 10.1016/j.psj.2025.105565 (PMC12296449; doi:10.1016/j.psj.2025.105565)

## Slide 1
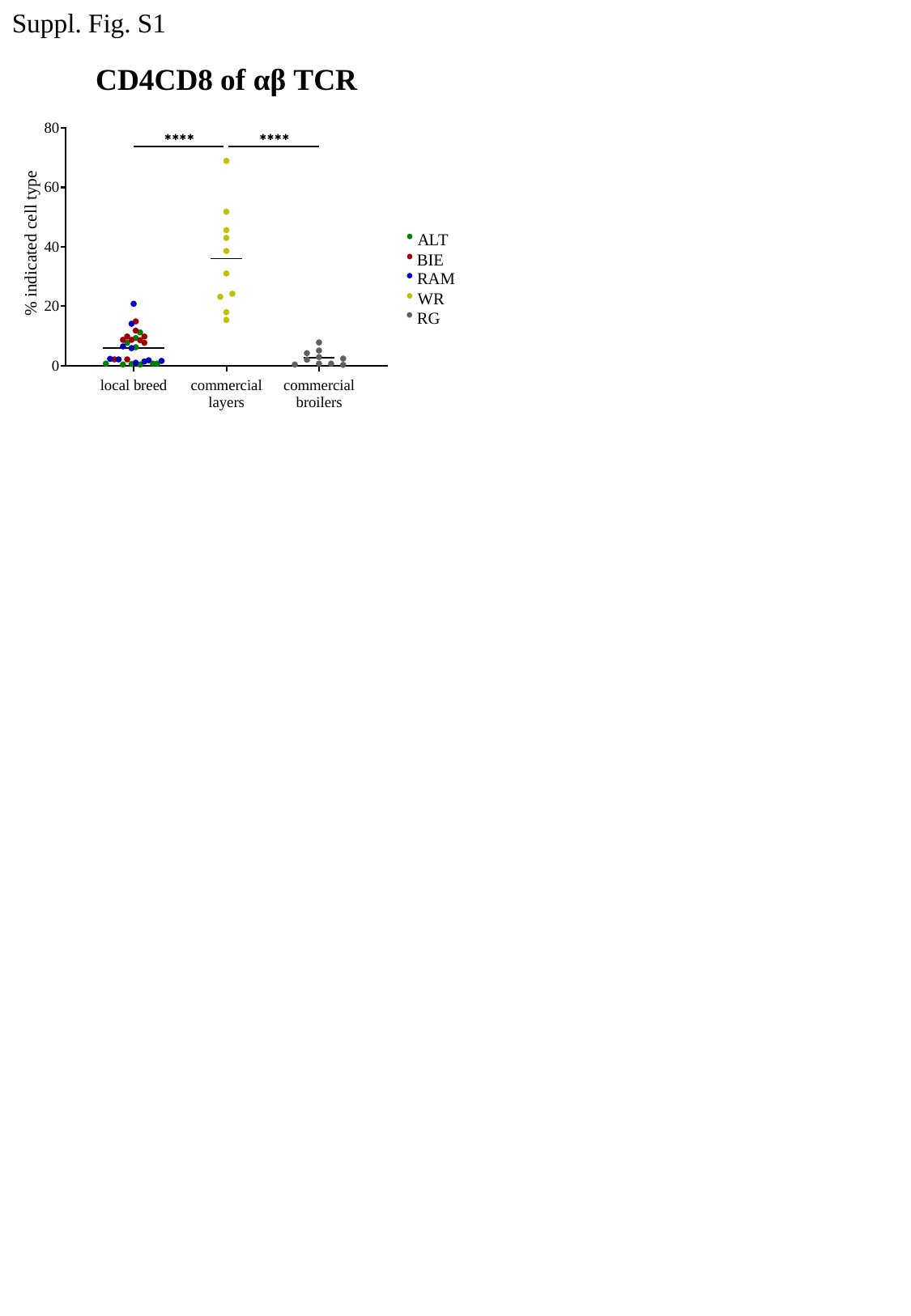

Suppl. Fig. S1

Supplement: Supplementary Fig. S1 — Levels of CD4+CD8+ double positive αβ TCR+ T cells in 35-week-old laying hens at peak performance, comparing local breeds and commercial lines. Significance levels were indicated as follows: P ≤ 0.0001 (****). TCR = T cell receptor, ALT = Altsteirer, BIE = Bielefelder, RAM = Ramelsloher, WR = White Rock, RG = Ranger. [file mmc1.pptx]
